# Schizophrenia, Depressive Symptoms, and Antipsychotic Drug Treatment

**DOI:** 10.1093/ijnp/pyaa091

**Published:** 2021-01-06

**Authors:** Gavin P Reynolds, Olga O McGowan

**Affiliations:** 1 Biomolecular Sciences Research Centre, Sheffield Hallam University, Sheffield, United Kingdom; 2 Gartnavel Royal Hospital, Glasgow, United Kingdom

Depressive symptoms are a common, if under-researched and poorly understood, feature of schizophrenia. They can occur at any stage of the illness, with a reported prevalence of approximately 40% (Conley et al., 2007). Depressive symptoms can be important indicators of outcome; they contribute to suicidality, and relapse can follow a deterioration in depressive (but not positive or negative) symptoms (Tollefson et al., 1999). It is perhaps unsurprising that low mood may be apparent in people experiencing unpleasant and frequently debilitating psychotic symptoms. Yet a depressed mood is more than just a consequence of psychosis; depressive symptoms can, for example, occur prior to the manifestation of positive psychotic symptoms (Yung et al., 2004).

These symptoms are, by definition, equivalent those typically presenting in major depressive disorder. They can include the full gamut of features, including apathy and social withdrawal as well as low mood, although some typical depressive symptoms such as weight loss or sleep disturbance may be confounded by drug treatment. It is also apparent that there may be overlap with the negative symptoms of schizophrenia, of which withdrawal and anhedonia are considered features. There is little to suggest that depressive symptoms of schizophrenia respond to antidepressant augmentation of antipsychotic drug treatment, although it may be beneficial for negative symptoms (Galling et al., 2018). This observation can be interpreted in 2 ways: either the antipsychotic drugs themselves have antidepressant action making further intervention ineffective, or the depressive symptoms in schizophrenia are inherently different from those that respond to such treatment in major depression. There is certainly strong evidence for the former explanation.

It is clear that antipsychotic treatments may have antidepressant effects; several antipsychotic drugs are now also licensed for use in unipolar as well as bipolar depression. Second-generation antipsychotics (SGAs) have some efficacy in treatment-resistant depression (Zhou et al., 2015); a recent Cochrane review indicated that augmentation of antidepressant therapy with cariprazine, olanzapine, quetiapine, or ziprasidone improved depressive symptoms in the short term (Davies et al., 2019).

It is thus unsurprising that some antipsychotics may relieve the depressive symptoms of schizophrenia. The study by Miura et al. (2020) addresses this with a meta-analysis of placebo-controlled trials in which they demonstrated that most of the commonly used SGAs had efficacy in relieving depressive symptoms, while the 2 first-generation drugs, haloperidol and chlorpromazine, showed no significant effect.

One anomaly was the result for ziprasidone, which they found had no significant influence on depressive symptoms over placebo. This may be a misleading finding reflective of the relatively small ziprasidone sample; the network meta-analysis of Huhn et al. (2019) suggests a significant if small effect in depressive symptom improvement for ziprasidone, albeit significantly weaker than olanzapine (and amisulpride and sulpiride, not assessed by Miura et al., 2020) and similar to lurasidone. Some further findings from the Huhn et al. (2019) analysis are worth contrasting with this study. Haloperidol was shown to be significantly more effective than placebo, which might argue against the conclusion that, of the antipsychotic drugs, it is only the SGAs that are effective in antidepressant action. Addington et al. (2011), in analyzing data from the CATIE study, also found no evidence for a class effect of SGAs by showing that perphenazine also had efficacy in ameliorating depressive symptoms.

What might be the underlying pharmacological basis of the antidepressant action of antipsychotic drugs? The 2 neurotransmitter systems that have been most implicated in depression and antidepressant response are those of serotonin and noradrenaline. SGAs do not share with the antidepressant drugs an action in inhibiting serotonin or noradrenaline reuptake. They do, however, have effects at various receptors for these transmitters, and it seems likely that such receptor effects are important in the antidepressant action of antipsychotic drugs.

There are several serotonin (5-hydroxytryptamine, 5-HT) receptors that may contribute to antidepressant activity. The 5-HT2A site is a good candidate; antagonism of these receptors, a feature of almost all SGAs, is thought to contribute to the ability of these drugs to augment the antidepressant effects of the SSRIs (Rogóż, 2013). However, this leaves unexplained the particular efficacy of amisulpride (Huhn et al., 2019), an antipsychotic without 5-HT2A receptor affinity.

Thus, other receptors are likely to be involved in differentiating the antipsychotics in their antidepressant efficacy. Two receptor mechanisms that may be important in this respect are alpha2 adrenergic receptor antagonism and 5-HT1A receptor partial agonism, both a feature of clozapine pharmacology. Antagonism of alpha2 adrenergic receptors increases synaptic serotonin and noradrenaline release, a presumed mode of action of the antidepressant mirtazapine, although the lack of efficacy of augmentation with this drug in relieving the depressive symptoms of schizophrenia (Kishi and Iwata, 2014), or in improving treatment-resistant depression as do some SGAs (Davies et al., 2019), might argue against this mechanism. However, an intriguing observation by Terevnikov et al. (2015) points to a noradrenergic mechanism. Among the adjunctive antidepressants studied in schizophrenia, only “imipramine and duloxetine tended to improve depressive symptoms.” Both of these drugs block reuptake of noradrenaline as well as of serotonin. While the antipsychotics themselves have no substantial effect on monoamine transmitter transporters, quetiapine’s major metabolite, norquetiapine, can inhibit noradrenergic reuptake (Cross et al., 2016), an effect likely to be clinically functional (Yatham et al., 2018). Nevertheless, Miura et al. (2020) do not demonstrate any advantage of quetiapine over other SGAs.

The 5-HT1A receptors regulate serotonin neuronal activity, and partial agonist activity at this site is a feature of some, but not all, SGAs. Other serotonin receptors have also been implicated, such as 5-HT7, at which amisulpride, lurasidone, and several other SGAs are antagonists. However, activity at any one particular site does not obviously correlate with relative antidepressant efficacy. Furthermore, it is hard to ignore the possible contribution of dopamine D2 receptor mechanisms, common to all antipsychotic drugs, if an antidepressant action of antipsychotics is not specific to any pharmacologically defined subgroup. Certainly the dopamine system is implicated in depression and antidepressant mechanisms. A rat model of stress-induced depression is associated with reduced activity of limbic dopaminergic neurons; this can be normalized by repeated administration of quetiapine at doses equivalent to those used for antidepressant treatment (Moreines et al., 2017), although the underlying receptor mechanism remains elusive.

Further clues relating to these various pharmacological mechanisms may come from studies of factors contributing to individual variability. It is clear that depressive symptoms do not consistently improve in all people with schizophrenia following antipsychotic drug treatment; some respond well, whereas some respond poorly or even deteriorate. Genetic variability is likely to contribute to these inter-individual differences. Reflecting the general paucity of research into depressive symptoms in schizophrenia, few pharmacogenetic studies have addressed their response to treatment. An early small investigation identified possible associations with genetic polymorphisms, including the alpha1A adrenoceptor and the 5-HT6 receptor (Houston et al., 2007); a larger study found association with a D2 receptor polymorphism (Misiak et al., 2016). However, there have been other robust findings. The 5-HT1A receptor gene has a functional polymorphism (rs6295) associated with depression and antidepressant response (Lemonde et al., 2004). A study of this polymorphism in antipsychotic drug response showed that it was associated, independently, with change in both negative and depressive symptoms in first-episode patients receiving risperidone or olanzapine (Reynolds et al., 2006). While the association of rs6295 with negative symptom response has been observed in several further studies (Takekita et al., 2016), 1 report has replicated this association with depressive symptom improvement with aripiprazole (Chen and Shen, 2017).

While these findings may tell us something about the biology underlying depressive, and negative, symptom response to treatment, they do indicate the lack of a clear biological distinction between the 2 symptom domains. This may be in part methodological; of the measures used for assessment of depressive symptoms in schizophrenia, only the Calgary Depression Scale is designed to differentiate these from the negative symptoms. Thus, for many analyses using less specific scales, such as the Hamilton Depression Rating Scale, changes in negative symptoms may confound the findings (Addington et al., 1996). It is notable that the Montgomery-Åsberg Depression Rating Scale (MADRS) scale, on which depressive symptoms of schizophrenia have been assessed in several of the studies included in the meta-analysis of Miura et al. (2020) contains several items that could be considered negative symptoms; MADRS is found to correlate with negative as well as depressive components of the Positive and Negative Syndrome Scale (Wolthaus et al., 2000).

However, depression in schizophrenia cannot be fully differentiated from negative symptoms (Krynicki et al., 2018). It may perhaps be worth considering that further attempts at the phenomenological division of distinct depressive and negative symptom domains might be less valuable than accepting they may have a common biological basis and that future research could benefit more from a search to understand the reasons why, for these symptoms, some patients respond better than others to drug treatments. Genetic studies provide just 1 approach towards this goal.

## Statement of Interest

G.P.R. has received honoraria for lectures from Kang Hong, Lundbeck, Otsuka, and Sumitomo. O.O.M. has no financial interest to declare.
